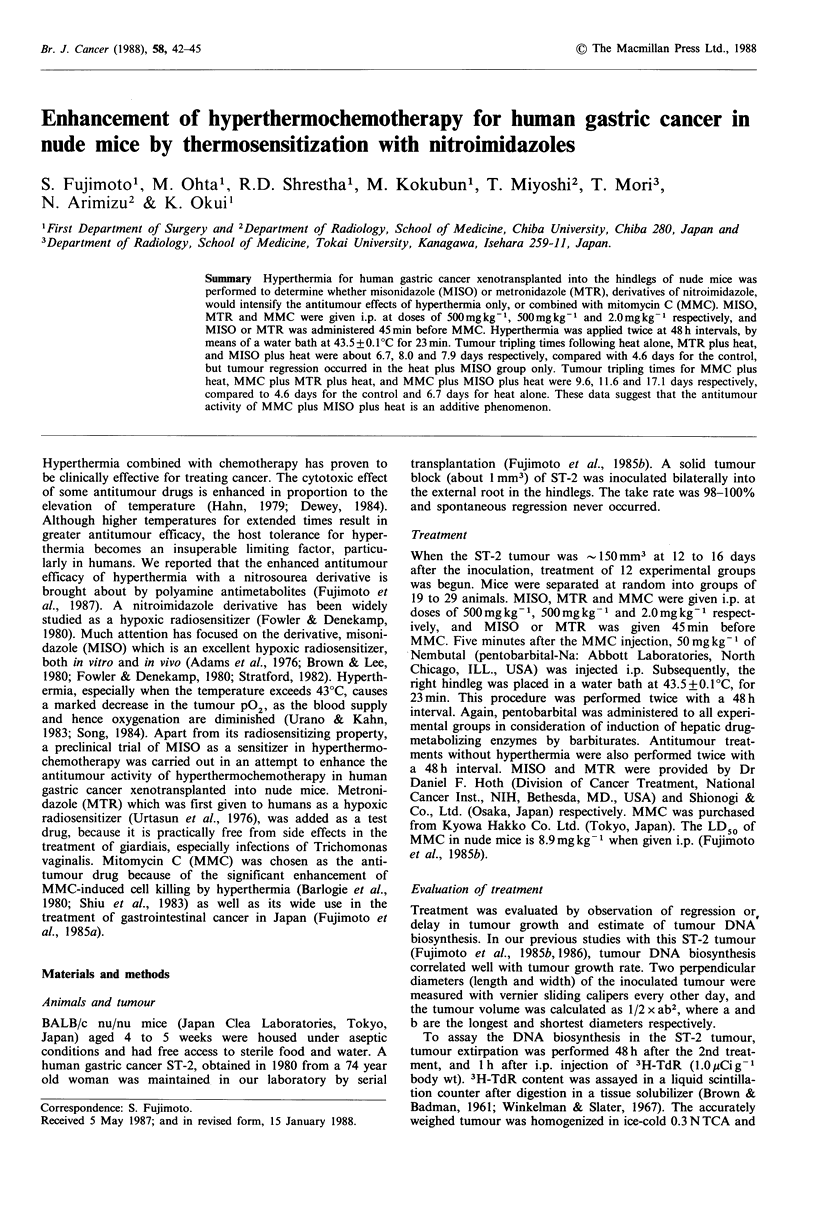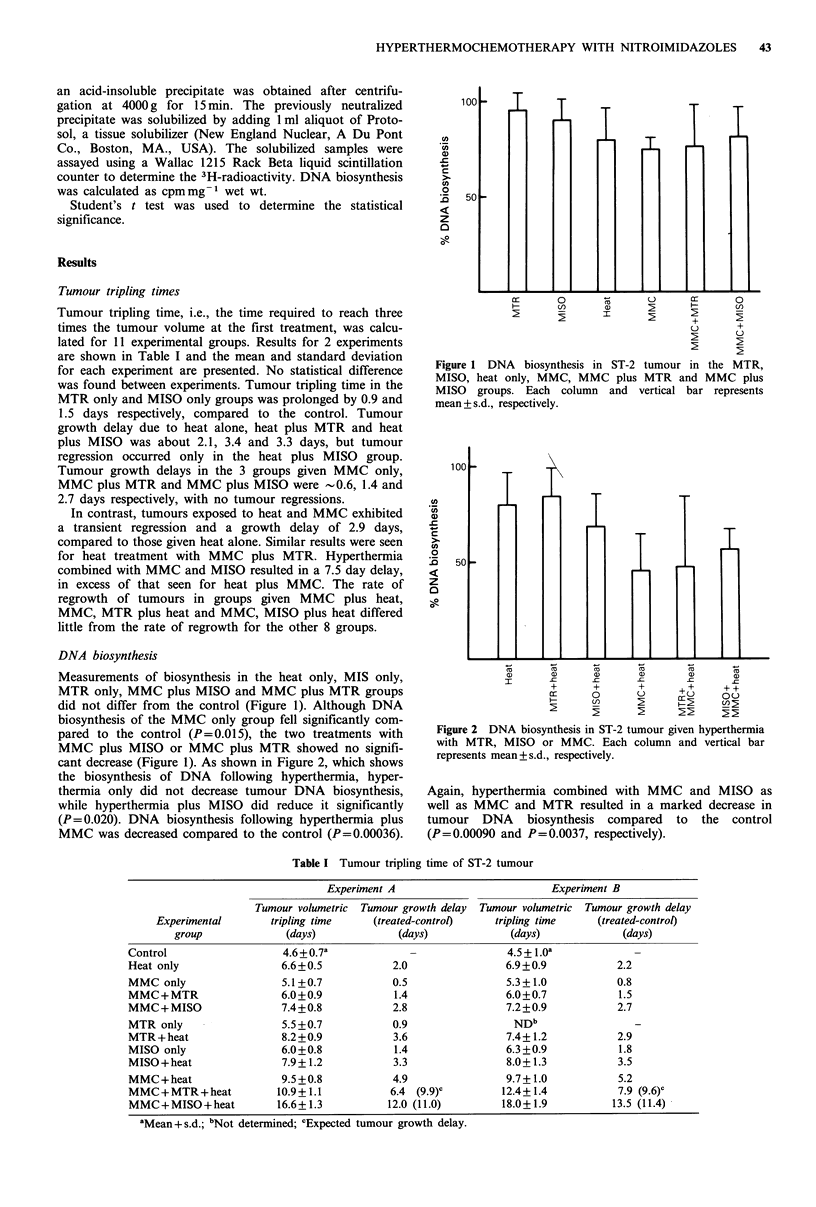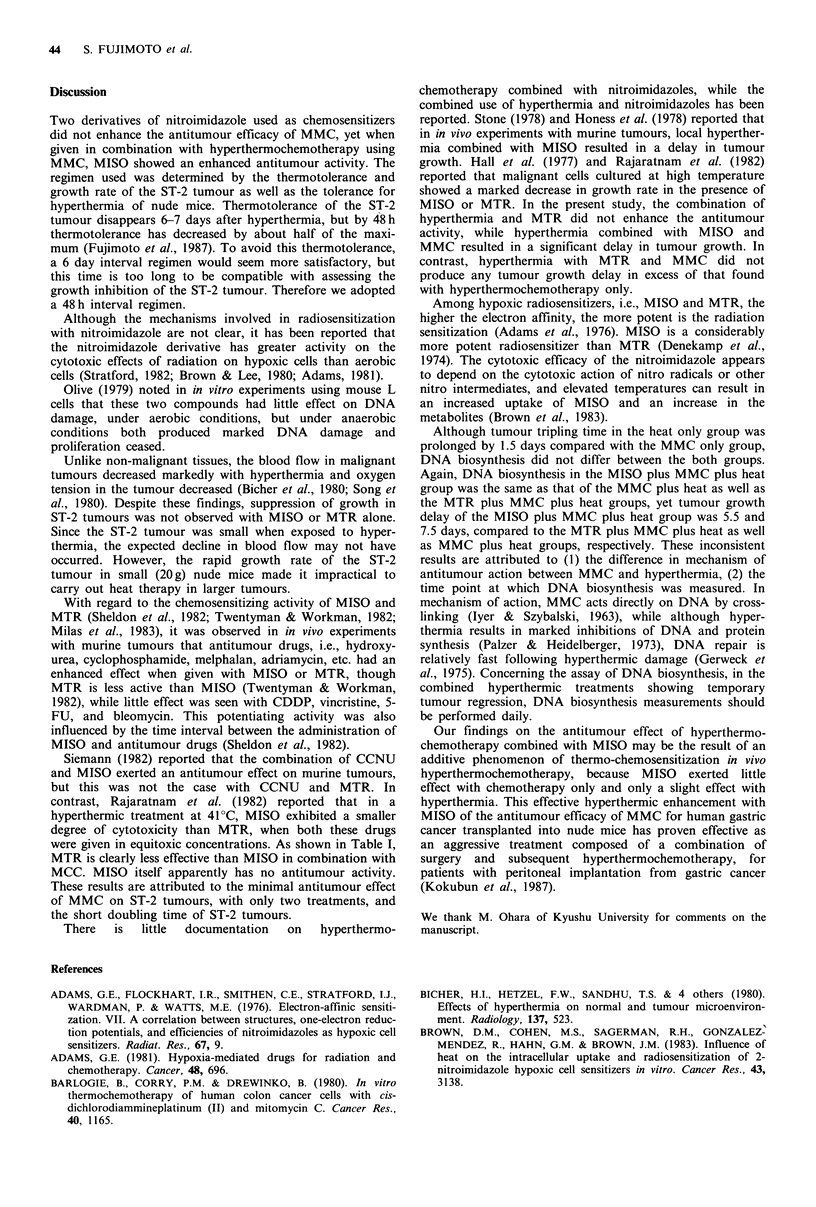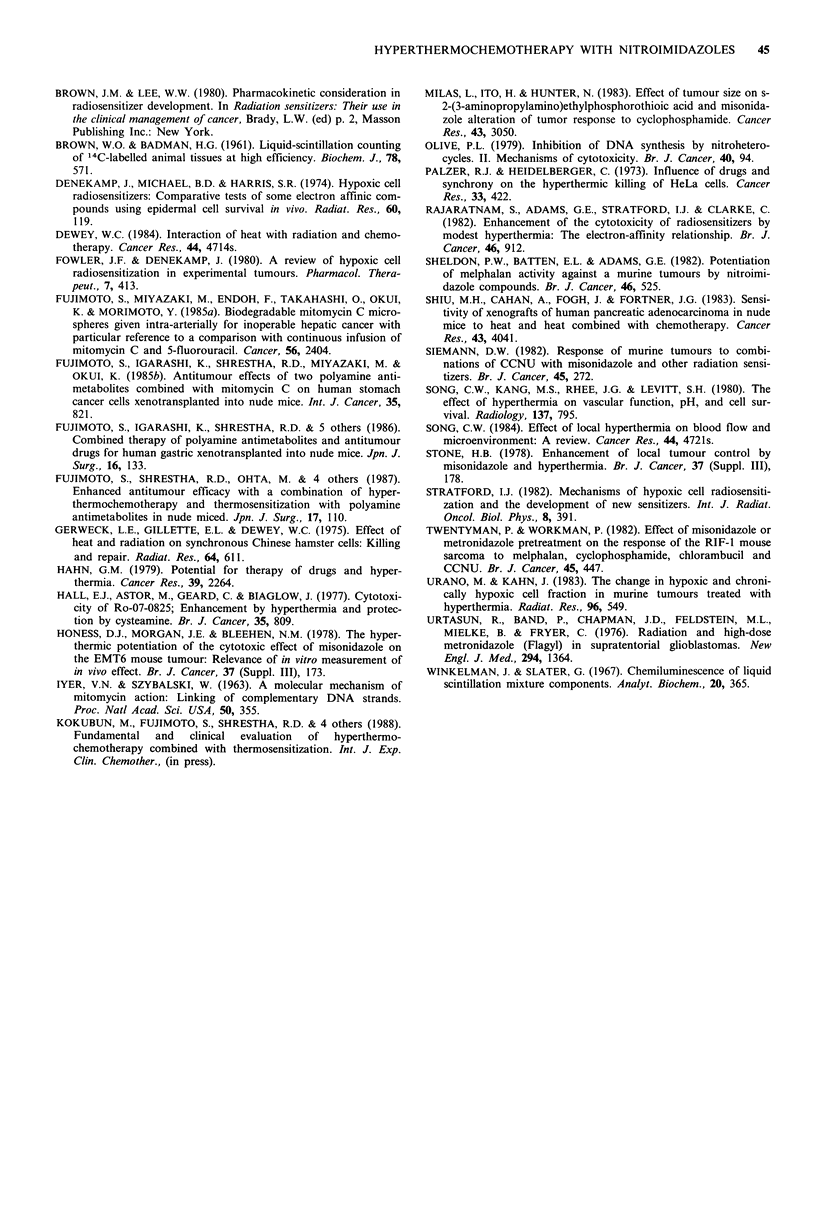# Enhancement of hyperthermochemotherapy for human gastric cancer in nude mice by thermosensitization with nitroimidazoles.

**DOI:** 10.1038/bjc.1988.158

**Published:** 1988-07

**Authors:** S. Fujimoto, M. Ohta, R. D. Shrestha, M. Kokubun, T. Miyoshi, T. Mori, N. Arimizu, K. Okui

**Affiliations:** First Department of Surgery, School of Medicine, Chiba University, Japan.

## Abstract

Hyperthermia for human gastric cancer xenotransplanted into the hindlegs of nude mice was performed to determine whether misonidazole (MISO) or metronidazole (MTR), derivatives of nitroimidazole, would intensify the antitumour effects of hyperthermia only, or combined with mitomycin C (MMC). MISO, MTR and MMC were given i.p. at doses of 500 mg kg-1, 500 mg kg-1 and 2.0 mg kg-1 respectively, and MISO or MTR was administered 45 min before MMC. Hyperthermia was applied twice at 48 h intervals, by means of a water bath at 43.5 +/- 0.1 degrees C for 23 min. Tumour tripling times following heat alone, MTR plus heat, and MISO plus heat were about 6.7, 8.0 and 7.9 days respectively, compared with 4.6 days for the control, but tumour regression occurred in the heat plus MISO group only. Tumour tripling times for MMC plus heat, MMC plus MTR plus heat, and MMC plus MISO plus heat were 9.6, 11.6 and 17.1 days respectively, compared to 4.6 days for the control and 6.7 days for heat alone. These data suggest that the antitumour activity of MMC plus MISO plus heat is an additive phenomenon.


*Br. J. Cancer* (1988), **58**, 42–45 © The Macmillan Press Ltd., 1988

### Enhancement of hyperthermochemotherapy for human gastric cancer in nude mice by thermosensitization with nitroimidazoles

S. Fujimoto[1], M. Ohta[1], R.D. Shrestha[1], M. Kokubun[1], T. Miyoshi[2], T. Mori[3], N. Arimizu[2] & K. Okui[1]

[1]*First Department of Surgery and* [2]*Department of Radiology, School of Medicine, Chiba University, Chiba 280, Japan and* [3]*Department of Radiology, School of Medicine, Tokai University, Kanagawa, Isehara 259-11, Japan.*

**Summary** Hyperthermia for human gastric cancer xenotransplanted into the hindlegs of nude mice was performed to determine whether misonidazole (MISO) or metronidazole (MTR), derivatives of nitroimidazole, would intensify the antitumour effects of hyperthermia only, or combined with mitomycin C (MMC). MISO, MTR and MMC were given i.p. at doses of $500 mg kg^{-1}$, $500 mg kg^{-1}$ and $2.0 mg kg^{-1}$ respectively, and MISO or MTR was administered 45 min before MMC. Hyperthermia was applied twice at 48 h intervals, by means of a water bath at $43.5 \pm 0.1$°C for 23 min. Tumour tripling times following heat alone, MTR plus heat, and MISO plus heat were about 6.7, 8.0 and 7.9 days respectively, compared with 4.6 days for the control, but tumour regression occurred in the heat plus MISO group only. Tumour tripling times for MMC plus heat, MMC plus MTR plus heat, and MMC plus MISO plus heat were 9.6, 11.6 and 17.1 days respectively, compared to 4.6 days for the control and 6.7 days for heat alone. These data suggest that the antitumour activity of MMC plus MISO plus heat is an additive phenomenon.

Hyperthermia combined with chemotherapy has proven to be clinically effective for treating cancer. The cytotoxic effect of some antitumour drugs is enhanced in proportion to the elevation of temperature (Hahn, 1979; Dewey, 1984). Although higher temperatures for extended times result in greater antitumour efficacy, the host tolerance for hyperthermia becomes an insuperable limiting factor, particularly in humans. We reported that the enhanced antitumour efficacy of hyperthermia with a nitrosourea derivative is brought about by polyamine antimetabolites (Fujimoto *et al.*, 1987). A nitroimidazole derivative has been widely studied as a hypoxic radiosensitizer (Fowler & Denekamp, 1980). Much attention has focused on the derivative, misonidazole (MISO) which is an excellent hypoxic radiosensitizer, both *in vitro* and *in vivo* (Adams *et al.*, 1976; Brown & Lee, 1980; Fowler & Denekamp, 1980; Stratford, 1982). Hyperthermia, especially when the temperature exceeds 43°C, causes a marked decrease in the tumour $pO_2$, as the blood supply and hence oxygenation are diminished (Urano & Kahn, 1983; Song, 1984). Apart from its radiosensitizing property, a preclinical trial of MISO as a sensitizer in hyperthermochemotherapy was carried out in an attempt to enhance the antitumour activity of hyperthermochemotherapy in human gastric cancer xenotransplanted into nude mice. Metronidazole (MTR) which was first given to humans as a hypoxic radiosensitizer (Urtasun *et al.*, 1976), was added as a test drug, because it is practically free from side effects in the treatment of giardiais, especially infections of Trichomonas vaginalis. Mitomycin C (MMC) was chosen as the antitumour drug because of the significant enhancement of MMC-induced cell killing by hyperthermia (Barlogie *et al.*, 1980; Shiu *et al.*, 1983) as well as its wide use in the treatment of gastrointestinal cancer in Japan (Fujimoto *et al.*, 1985*a*).

### Materials and methods

### *Animals and tumour*

BALB/c nu/nu mice (Japan Clea Laboratories, Tokyo, Japan) aged 4 to 5 weeks were housed under aseptic conditions and had free access to sterile food and water. A human gastric cancer ST-2, obtained in 1980 from a 74 year old woman was maintained in our laboratory by serial transplantation (Fujimoto *et al.*, 1985*b*). A solid tumour block (about $1 mm^3$) of ST-2 was inoculated bilaterally into the external root in the hindlegs. The take rate was 98–100% and spontaneous regression never occurred.

### *Treatment*

When the ST-2 tumour was $\sim 150 mm^3$ at 12 to 16 days after the inoculation, treatment of 12 experimental groups was begun. Mice were separated at random into groups of 19 to 29 animals. MISO, MTR and MMC were given i.p. at doses of $500 mg kg^{-1}$, $500 mg kg^{-1}$ and $2.0 mg kg^{-1}$ respectively, and MISO or MTR was given 45 min before MMC. Five minutes after the MMC injection, $50 mg kg^{-1}$ of Nembutal (pentobarbital-Na: Abbott Laboratories, North Chicago, ILL., USA) was injected i.p. Subsequently, the right hindleg was placed in a water bath at $43.5 \pm 0.1$°C, for 23 min. This procedure was performed twice with a 48 h interval. Again, pentobarbital was administered to all experimental groups in consideration of induction of hepatic drug-metabolizing enzymes by barbiturates. Antitumour treatments without hyperthermia were also performed twice with a 48 h interval. MISO and MTR were provided by Dr Daniel F. Hoth (Division of Cancer Treatment, National Cancer Inst., NIH, Bethesda, MD., USA) and Shionogi & Co., Ltd. (Osaka, Japan) respectively. MMC was purchased from Kyowa Hakko Co. Ltd. (Tokyo, Japan). The $LD_{50}$ of MMC in nude mice is $8.9 mg kg^{-1}$ when given i.p. (Fujimoto *et al.*, 1985*b*).

### *Evaluation of treatment*

Treatment was evaluated by observation of regression or delay in tumour growth and estimate of tumour DNA biosynthesis. In our previous studies with this ST-2 tumour (Fujimoto *et al.*, 1985*b*, 1986), tumour DNA biosynthesis correlated well with tumour growth rate. Two perpendicular diameters (length and width) of the inoculated tumour were measured with vernier sliding calipers every other day, and the tumour volume was calculated as $1/2 \times ab^2$, where a and b are the longest and shortest diameters respectively.

To assay the DNA biosynthesis in the ST-2 tumour, tumour extirpation was performed 48 h after the 2nd treatment, and 1 h after i.p. injection of $^3$H-TdR ($1.0 \mu Ci g^{-1}$ body wt). $^3$H-TdR content was assayed in a liquid scintillation counter after digestion in a tissue solubilizer (Brown & Badman, 1961; Winkelman & Slater, 1967). The accurately weighed tumour was homogenized in ice-cold 0.3 N TCA and

Correspondence: S. Fujimoto.
Received 5 May 1987; and in revised form, 15 January 1988.

an acid-insoluble precipitate was obtained after centrifugation at 4000 g for 15 min. The previously neutralized precipitate was solubilized by adding 1 ml aliquot of Protosol, a tissue solubilizer (New England Nuclear, A Du Pont Co., Boston, MA., USA). The solubilized samples were assayed using a Wallac 1215 Rack Beta liquid scintillation counter to determine the $^{3}$H-radioactivity. DNA biosynthesis was calculated as cpm mg$^{-1}$ wet wt.

Student's *t* test was used to determine the statistical significance.

### Results

### *Tumour tripling times*

Tumour tripling time, i.e., the time required to reach three times the tumour volume at the first treatment, was calculated for 11 experimental groups. Results for 2 experiments are shown in Table I and the mean and standard deviation for each experiment are presented. No statistical difference was found between experiments. Tumour tripling time in the MTR only and MISO only groups was prolonged by 0.9 and 1.5 days respectively, compared to the control. Tumour growth delay due to heat alone, heat plus MTR and heat plus MISO was about 2.1, 3.4 and 3.3 days, but tumour regression occurred only in the heat plus MISO group. Tumour growth delays in the 3 groups given MMC only, MMC plus MTR and MMC plus MISO were ~0.6, 1.4 and 2.7 days respectively, with no tumour regressions.

In contrast, tumours exposed to heat and MMC exhibited a transient regression and a growth delay of 2.9 days, compared to those given heat alone. Similar results were seen for heat treatment with MMC plus MTR. Hyperthermia combined with MMC and MISO resulted in a 7.5 day delay, in excess of that seen for heat plus MMC. The rate of regrowth of tumours in groups given MMC plus heat, MMC, MTR plus heat and MMC, MISO plus heat differed little from the rate of regrowth for the other 8 groups.

### *DNA biosynthesis*

Measurements of biosynthesis in the heat only, MIS only, MTR only, MMC plus MISO and MMC plus MTR groups did not differ from the control (Figure 1). Although DNA biosynthesis of the MMC only group fell significantly compared to the control ($P=0.015$), the two treatments with MMC plus MISO or MMC plus MTR showed no significant decrease (Figure 1). As shown in Figure 2, which shows the biosynthesis of DNA following hyperthermia, hyperthermia only did not decrease tumour DNA biosynthesis, while hyperthermia plus MISO did reduce it significantly ($P=0.020$). DNA biosynthesis following hyperthermia plus MMC was decreased compared to the control ($P=0.00036$).

**Figure 1** DNA biosynthesis in ST-2 tumour in the MTR, MISO, heat only, MMC, MMC plus MTR and MMC plus MISO groups. Each column and vertical bar represents mean ± s.d., respectively.

**Figure 2** DNA biosynthesis in ST-2 tumour given hyperthermia with MTR, MISO or MMC. Each column and vertical bar represents mean ± s.d., respectively.

Again, hyperthermia combined with MMC and MISO as well as MMC and MTR resulted in a marked decrease in tumour DNA biosynthesis compared to the control ($P=0.00090$ and $P=0.0037$, respectively).

**Table I** Tumour tripling time of ST-2 tumour

| | *Experiment A* | | *Experiment B* | |
|---|---|---|---|---|
| *Experimental group* | *Tumour volumetric tripling time (days)* | *Tumour growth delay (treated-control) (days)* | *Tumour volumetric tripling time (days)* | *Tumour growth delay (treated-control) (days)* |
| Control | 4.6±0.7[a] | – | 4.5±1.0[a] | – |
| Heat only | 6.6±0.5 | 2.0 | 6.9±0.9 | 2.2 |
| MMC only | 5.1±0.7 | 0.5 | 5.3±1.0 | 0.8 |
| MMC+MTR | 6.0±0.9 | 1.4 | 6.0±0.7 | 1.5 |
| MMC+MISO | 7.4±0.8 | 2.8 | 7.2±0.9 | 2.7 |
| MTR only | 5.5±0.7 | 0.9 | ND[b] | – |
| MTR+heat | 8.2±0.9 | 3.6 | 7.4±1.2 | 2.9 |
| MISO only | 6.0±0.8 | 1.4 | 6.3±0.9 | 1.8 |
| MISO+heat | 7.9±1.2 | 3.3 | 8.0±1.3 | 3.5 |
| MMC+heat | 9.5±0.8 | 4.9 | 9.7±1.0 | 5.2 |
| MMC+MTR+heat | 10.9±1.1 | 6.4 (9.9)[c] | 12.4±1.4 | 7.9 (9.6)[c] |
| MMC+MISO+heat | 16.6±1.3 | 12.0 (11.0) | 18.0±1.9 | 13.5 (11.4) |

[a]Mean+s.d.; [b]Not determined; [c]Expected tumour growth delay.

### Discussion

Two derivatives of nitroimidazole used as chemosensitizers did not enhance the antitumour efficacy of MMC, yet when given in combination with hyperthermochemotherapy using MMC, MISO showed an enhanced antitumour activity. The regimen used was determined by the thermotolerance and growth rate of the ST-2 tumour as well as the tolerance for hyperthermia of nude mice. Thermotolerance of the ST-2 tumour disappears 6–7 days after hyperthermia, but by 48 h thermotolerance has decreased by about half of the maximum (Fujimoto *et al.*, 1987). To avoid this thermotolerance, a 6 day interval regimen would seem more satisfactory, but this time is too long to be compatible with assessing the growth inhibition of the ST-2 tumour. Therefore we adopted a 48 h interval regimen.

Although the mechanisms involved in radiosensitization with nitroimidazole are not clear, it has been reported that the nitroimidazole derivative has greater activity on the cytotoxic effects of radiation on hypoxic cells than aerobic cells (Stratford, 1982; Brown & Lee, 1980; Adams, 1981).

Olive (1979) noted in *in vitro* experiments using mouse L cells that these two compounds had little effect on DNA damage, under aerobic conditions, but under anaerobic conditions both produced marked DNA damage and proliferation ceased.

Unlike non-malignant tissues, the blood flow in malignant tumours decreased markedly with hyperthermia and oxygen tension in the tumour decreased (Bicher *et al.*, 1980; Song *et al.*, 1980). Despite these findings, suppression of growth in ST-2 tumours was not observed with MISO or MTR alone. Since the ST-2 tumour was small when exposed to hyperthermia, the expected decline in blood flow may not have occurred. However, the rapid growth rate of the ST-2 tumour in small (20 g) nude mice made it impractical to carry out heat therapy in larger tumours.

With regard to the chemosensitizing activity of MISO and MTR (Sheldon *et al.*, 1982; Twentyman & Workman, 1982; Milas *et al.*, 1983), it was observed in *in vivo* experiments with murine tumours that antitumour drugs, i.e., hydroxyurea, cyclophosphamide, melphalan, adriamycin, etc. had an enhanced effect when given with MISO or MTR, though MTR is less active than MISO (Twentyman & Workman, 1982), while little effect was seen with CDDP, vincristine, 5-FU, and bleomycin. This potentiating activity was also influenced by the time interval between the administration of MISO and antitumour drugs (Sheldon *et al.*, 1982).

Siemann (1982) reported that the combination of CCNU and MISO exerted an antitumour effect on murine tumours, but this was not the case with CCNU and MTR. In contrast, Rajaratnam *et al.* (1982) reported that in a hyperthermic treatment at 41°C, MISO exhibited a smaller degree of cytotoxicity than MTR, when both these drugs were given in equitoxic concentrations. As shown in Table I, MTR is clearly less effective than MISO in combination with MCC. MISO itself apparently has no antitumour activity. These results are attributed to the minimal antitumour effect of MMC on ST-2 tumours, with only two treatments, and the short doubling time of ST-2 tumours.

There is little documentation on hyperthermochemotherapy combined with nitroimidazoles, while the combined use of hyperthermia and nitroimidazoles has been reported. Stone (1978) and Honess *et al.* (1978) reported that in *in vivo* experiments with murine tumours, local hyperthermia combined with MISO resulted in a delay in tumour growth. Hall *et al.* (1977) and Rajaratnam *et al.* (1982) reported that malignant cells cultured at high temperature showed a marked decrease in growth rate in the presence of MISO or MTR. In the present study, the combination of hyperthermia and MTR did not enhance the antitumour activity, while hyperthermia combined with MISO and MMC resulted in a significant delay in tumour growth. In contrast, hyperthermia with MTR and MMC did not produce any tumour growth delay in excess of that found with hyperthermochemotherapy only.

Among hypoxic radiosensitizers, i.e., MISO and MTR, the higher the electron affinity, the more potent is the radiation sensitization (Adams *et al.*, 1976). MISO is a considerably more potent radiosensitizer than MTR (Denekamp *et al.*, 1974). The cytotoxic efficacy of the nitroimidazole appears to depend on the cytotoxic action of nitro radicals or other nitro intermediates, and elevated temperatures can result in an increased uptake of MISO and an increase in the metabolites (Brown *et al.*, 1983).

Although tumour tripling time in the heat only group was prolonged by 1.5 days compared with the MMC only group, DNA biosynthesis did not differ between the both groups. Again, DNA biosynthesis in the MISO plus MMC plus heat group was the same as that of the MMC plus heat as well as the MTR plus MMC plus heat groups, yet tumour growth delay of the MISO plus MMC plus heat group was 5.5 and 7.5 days, compared to the MTR plus MMC plus heat as well as MMC plus heat groups, respectively. These inconsistent results are attributed to (1) the difference in mechanism of antitumour action between MMC and hyperthermia, (2) the time point at which DNA biosynthesis was measured. In mechanism of action, MMC acts directly on DNA by cross-linking (Iyer & Szybalski, 1963), while although hyperthermia results in marked inhibitions of DNA and protein synthesis (Palzer & Heidelberger, 1973), DNA repair is relatively fast following hyperthermic damage (Gerweck *et al.*, 1975). Concerning the assay of DNA biosynthesis, in the combined hyperthermic treatments showing temporary tumour regression, DNA biosynthesis measurements should be performed daily.

Our findings on the antitumour effect of hyperthermochemotherapy combined with MISO may be the result of an additive phenomenon of thermo-chemosensitization *in vivo* hyperthermochemotherapy, because MISO exerted little effect with chemotherapy only and only a slight effect with hyperthermia. This effective hyperthermic enhancement with MISO of the antitumour efficacy of MMC for human gastric cancer transplanted into nude mice has proven effective as an aggressive treatment composed of a combination of surgery and subsequent hyperthermochemotherapy, for patients with peritoneal implantation from gastric cancer (Kokubun *et al.*, 1987).

We thank M. Ohara of Kyushu University for comments on the manuscript.

### References

ADAMS, G.E., FLOCKHART, I.R., SMITHEN, C.E., STRATFORD, I.J., WARDMAN, P. & WATTS, M.E. (1976). Electron-affinic sensitization. VII. A correlation between structures, one-electron reduction potentials, and efficiencies of nitroimidazoles as hypoxic cell sensitizers. *Radiat. Res.*, **67**, 9.

ADAMS, G.E. (1981). Hypoxia-mediated drugs for radiation and chemotherapy. *Cancer*, **48**, 696.

BARLOGIE, B., CORRY, P.M. & DREWINKO, B. (1980). *In vitro* thermochemotherapy of human colon cancer cells with *cis*-dichlorodiammineplatinum (II) and mitomycin C. *Cancer Res.*, **40**, 1165.

BICHER, H.I., HETZEL, F.W., SANDHU, T.S. & 4 others (1980). Effects of hyperthermia on normal and tumour microenvironment. *Radiology*, **137**, 523.

BROWN, D.M., COHEN, M.S., SAGERMAN, R.H., GONZALEZ-MENDEZ, R., HAHN, G.M. & BROWN, J.M. (1983). Influence of heat on the intracellular uptake and radiosensitization of 2-nitroimidazole hypoxic cell sensitizers *in vitro*. *Cancer Res.*, **43**, 3138.

BROWN, J.M. & LEE, W.W. (1980). Pharmacokinetic consideration in radiosensitizer development. In *Radiation sensitizers: Their use in the clinical management of cancer,* Brady, L.W. (ed) p. 2, Masson Publishing Inc.: New York.

BROWN, W.O. & BADMAN, H.G. (1961). Liquid-scintillation counting of $^{14}$C-labelled animal tissues at high efficiency. *Biochem. J.,* **78,** 571.

DENEKAMP, J., MICHAEL, B.D. & HARRIS, S.R. (1974). Hypoxic cell radiosensitizers: Comparative tests of some electron affinic compounds using epidermal cell survival *in vivo. Radiat. Res.,* **60,** 119.

DEWEY, W.C. (1984). Interaction of heat with radiation and chemotherapy. *Cancer Res.,* **44,** 4714s.

FOWLER, J.F. & DENEKAMP, J. (1980). A review of hypoxic cell radiosensitization in experimental tumours. *Pharmacol. Therapeut.,* **7,** 413.

FUJIMOTO, S., MIYAZAKI, M., ENDOH, F., TAKAHASHI, O., OKUI, K. & MORIMOTO, Y. (1985*a*). Biodegradable mitomycin C microspheres given intra-arterially for inoperable hepatic cancer with particular reference to a comparison with continuous infusion of mitomycin C and 5-fluorouracil. *Cancer,* **56,** 2404.

FUJIMOTO, S., IGARASHI, K., SHRESTHA, R.D., MIYAZAKI, M. & OKUI, K. (1985*b*). Antitumour effects of two polyamine antimetabolites combined with mitomycin C on human stomach cancer cells xenotransplanted into nude mice. *Int. J. Cancer,* **35,** 821.

FUJIMOTO, S., IGARASHI, K., SHRESTHA, R.D. & 5 others (1986). Combined therapy of polyamine antimetabolites and antitumour drugs for human gastric xenotransplanted into nude mice. *Jpn. J. Surg.,* **16,** 133.

FUJIMOTO, S., SHRESTHA, R.D., OHTA, M. & 4 others (1987). Enhanced antitumour efficacy with a combination of hyperthermochemotherapy and thermosensitization with polyamine antimetabolites in nude miced. *Jpn. J. Surg.,* **17,** 110.

GERWECK, L.E., GILLETTE, E.L. & DEWEY, W.C. (1975). Effect of heat and radiation on synchronous Chinese hamster cells: Killing and repair. *Radiat. Res.,* **64,** 611.

HAHN, G.M. (1979). Potential for therapy of drugs and hyperthermia. *Cancer Res.,* **39,** 2264.

HALL, E.J., ASTOR, M., GEARD, C. & BIAGLOW, J. (1977). Cytotoxicity of Ro-07-0825; Enhancement by hyperthermia and protection by cysteamine. *Br. J. Cancer,* **35,** 809.

HONESS, D.J., MORGAN, J.E. & BLEEHEN, N.M. (1978). The hyperthermic potentiation of the cytotoxic effect of misonidazole on the EMT6 mouse tumour: Relevance of *in vitro* measurement of *in vivo* effect. *Br. J. Cancer,* **37** (Suppl. III), 173.

IYER, V.N. & SZYBALSKI, W. (1963). A molecular mechanism of mitomycin action: Linking of complementary DNA strands. *Proc. Natl Acad. Sci. USA,* **50,** 355.

KOKUBUN, M., FUJIMOTO, S., SHRESTHA, R.D. & 4 others (1988). Fundamental and clinical evaluation of hyperthermochemotherapy combined with thermosensitization. *Int. J. Exp. Clin. Chemother.,* (in press).

MILAS, L., ITO, H. & HUNTER, N. (1983). Effect of tumour size on s-2-(3-aminopropylamino)ethylphosphorothioic acid and misonidazole alteration of tumor response to cyclophosphamide. *Cancer Res.,* **43,** 3050.

OLIVE, P.L. (1979). Inhibition of DNA synthesis by nitroheterocycles. II. Mechanisms of cytotoxicity. *Br. J. Cancer,* **40,** 94.

PALZER, R.J. & HEIDELBERGER, C. (1973). Influence of drugs and synchrony on the hyperthermic killing of HeLa cells. *Cancer Res.,* **33,** 422.

RAJARATNAM, S., ADAMS, G.E., STRATFORD, I.J. & CLARKE, C. (1982). Enhancement of the cytotoxicity of radiosensitizers by modest hyperthermia: The electron-affinity relationship. *Br. J. Cancer,* **46,** 912.

SHELDON, P.W., BATTEN, E.L. & ADAMS, G.E. (1982). Potentiation of melphalan activity against a murine tumours by nitroimidazole compounds. *Br. J. Cancer,* **46,** 525.

SHIU, M.H., CAHAN, A., FOGH, J. & FORTNER, J.G. (1983). Sensitivity of xenografts of human pancreatic adenocarcinoma in nude mice to heat and heat combined with chemotherapy. *Cancer Res.,* **43,** 4041.

SIEMANN, D.W. (1982). Response of murine tumours to combinations of CCNU with misonidazole and other radiation sensitizers. *Br. J. Cancer,* **45,** 272.

SONG, C.W., KANG, M.S., RHEE, J.G. & LEVITT, S.H. (1980). The effect of hyperthermia on vascular function, pH, and cell survival. *Radiology,* **137,** 795.

SONG, C.W. (1984). Effect of local hyperthermia on blood flow and microenvironment: A review. *Cancer Res.,* **44,** 4721s.

STONE, H.B. (1978). Enhancement of local tumour control by misonidazole and hyperthermia. *Br. J. Cancer,* **37** (Suppl. III), 178.

STRATFORD, I.J. (1982). Mechanisms of hypoxic cell radiosensitization and the development of new sensitizers. *Int. J. Radiat. Oncol. Biol. Phys.,* **8,** 391.

TWENTYMAN, P. & WORKMAN, P. (1982). Effect of misonidazole or metronidazole pretreatment on the response of the RIF-1 mouse sarcoma to melphalan, cyclophosphamide, chlorambucil and CCNU. *Br. J. Cancer,* **45,** 447.

URANO, M. & KAHN, J. (1983). The change in hypoxic and chronically hypoxic cell fraction in murine tumours treated with hyperthermia. *Radiat. Res.,* **96,** 549.

URTASUN, R., BAND, P., CHAPMAN, J.D., FELDSTEIN, M.L., MIELKE, B. & FRYER, C. (1976). Radiation and high-dose metronidazole (Flagyl) in supratentorial glioblastomas. *New Engl. J. Med.,* **294,** 1364.

WINKELMAN, J. & SLATER, G. (1967). Chemiluminescence of liquid scintillation mixture components. *Analyt. Biochem.,* **20,** 365.